# A Look into *Bunyavirales* Genomes: Functions of Non-Structural (NS) Proteins

**DOI:** 10.3390/v13020314

**Published:** 2021-02-18

**Authors:** Shanna S. Leventhal, Drew Wilson, Heinz Feldmann, David W. Hawman

**Affiliations:** Laboratory of Virology, Rocky Mountain Laboratories, Division of Intramural Research, National Institute of Allergy and Infectious Diseases, National Institutes of Health, Hamilton, MT 59840, USA; shanna.leventhal@nih.gov (S.S.L.); drew.wilson@nih.gov (D.W.); feldmannh@niaid.nih.gov (H.F.)

**Keywords:** bunyavirales, non-structural proteins, peribunyaviridae, nairoviridae, hantaviridae, phenuiviridae, interferon antagonist

## Abstract

In 2016, the *Bunyavirales* order was established by the International Committee on Taxonomy of Viruses (ICTV) to incorporate the increasing number of related viruses across 13 viral families. While diverse, four of the families (*Peribunyaviridae, Nairoviridae, Hantaviridae, and Phenuiviridae*) contain known human pathogens and share a similar tri-segmented, negative-sense RNA genomic organization. In addition to the nucleoprotein and envelope glycoproteins encoded by the small and medium segments, respectively, many of the viruses in these families also encode for non-structural (NS) NSs and NSm proteins. The NSs of *Phenuiviridae* is the most extensively studied as a host interferon antagonist, functioning through a variety of mechanisms seen throughout the other three families. In addition, functions impacting cellular apoptosis, chromatin organization, and transcriptional activities, to name a few, are possessed by NSs across the families. *Peribunyaviridae*, *Nairoviridae*, and *Phenuiviridae* also encode an NSm, although less extensively studied than NSs, that has roles in antagonizing immune responses, promoting viral assembly and infectivity, and even maintenance of infection in host mosquito vectors. Overall, the similar and divergent roles of NS proteins of these human pathogenic *Bunyavirales* are of particular interest in understanding disease progression, viral pathogenesis, and developing strategies for interventions and treatments.

## 1. Introduction

**Figure 1:**
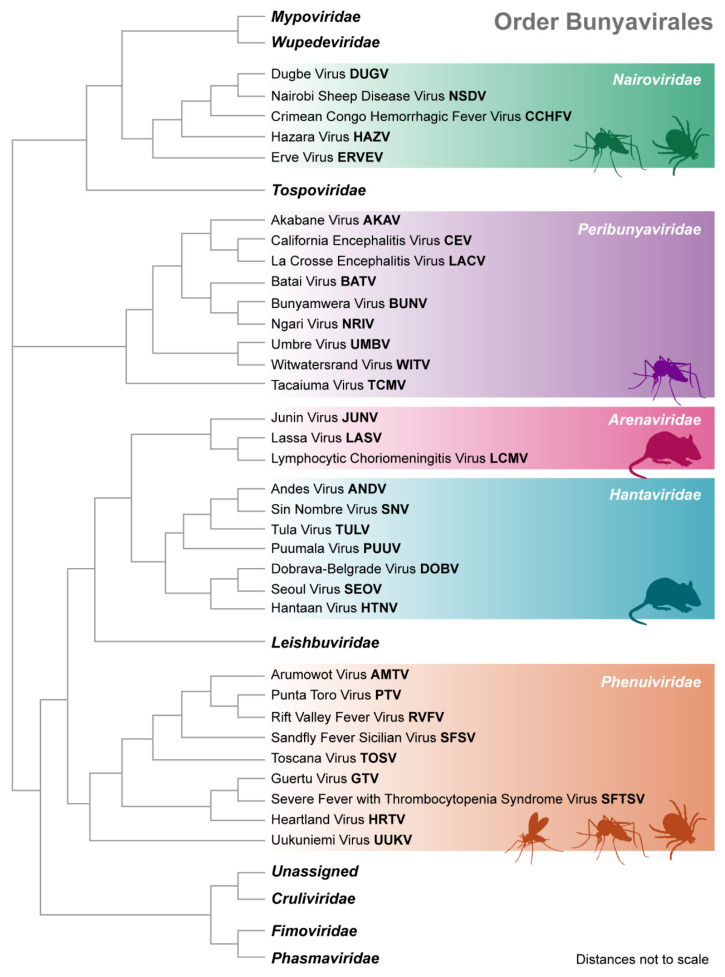
*Bunyavirales* Phylogenetic Tree. Order *Bunyavirales* phylogenetic tree based on nucleoprotein amino acid sequences of the 13 families. Families for which a specific virus is not listed are arranged via analysis of type species as listed in Table 1. Specific vector species are depicted for *Nairoviridae* (mosquito, tick), *Peribunyaviridae* (mosquito), *Arenaviridae* (rodent), *Hantaviridae* (rodent), and *Phenuiviridae* (sandfly, mosquito, tick). While these families may contain viruses transmitted through other vectors, these illustrations represent those of the viruses listed and discussed in this review. The phylogenetic tree was constructed using S segment nucleoprotein amino acid sequences from Genbank and was assembled using Geneious Prime tree builder global alignment with free end gaps, cost matrix PAM250, genetic distance model Jukes-Cantor, tree build method neighbor-joining, and no outgroup. The resulting tree was transformed so that branches are of equal length. Tree segments are not to scale. Genbank accession numbers for sequences used: *Mypoviridae* (NC_033760.1), *Wupedeviridae* (NC_043501.1), *Nairoviridae* (MH483984.1, FJ422213.2, MH791451.1, NC_038711.1, JF911699.1), *Tospoviridae* (MN861976.1), *Peribunyaviridae* (MH484290.1, MT276603.1, MH830340.1, MT022508.1, KM507341.1, MK896460.1, MK330166.1, NC_043673.1, LC552050.1), *Arenaviridae* (MG554174.1, MG189700.1, MT861994.1), *Hantaviridae* (MN258229.1, MT514275.1, MN832781.1, MN657233.1, MK360773.1, MT012546.1, KT885046.1), *Leishbuviridae* (KX280017.1), *Phenuiviridae* (DQ380149.1, NC_018137.1, NC_024496.1, EF201835.1, MT032306.1, KM114248.1, NC_043610.1, HM566145.1, EF201822.1), *Cruliviridae* (NC_032145.1), *Fimoviridae* (LR536377.1), *Phasmaviridae* (NC_043032.1), and *Unassigned* (MG764564.1).

In 1975, a group of serologically related, tri-segmented, negative-sense RNA viruses were grouped together by the International Committee on Taxonomy of Viruses (ICTV) as the *Bunyaviridae* family [1]. In 2016, the ICTV elevated *Bunyaviridae* to an order, *Bunyavirales*, to incorporate the increasing number of related viruses and new family classifications [1]. As of May 2019, the order contains 13 families (Figure 1, Table 1) whose viruses infect a variety of plants, animals, and insects, including *Cruliviridae* (crustaceans), *Fimoviridae* (plants), *Leishbuviridae* (parasites), *Mypoviridae* (insects), *Phasmaviridae* (insects), *Tospoviridae* (plants), *Wupedeviridae* (millipedes), and an *Unassigned* family containing a citrus tree bunyavirus [2]. Further, five families (*Arenaviridae*, *Peribunyaviridae*, *Nairoviridae*, *Phenuiviridae*, and *Hantaviridae*) contain notable human pathogens that cause mild to severe disease, including fevers, hemorrhagic disease, encephalitis, and respiratory disease [2,3].

**Figure 2:**
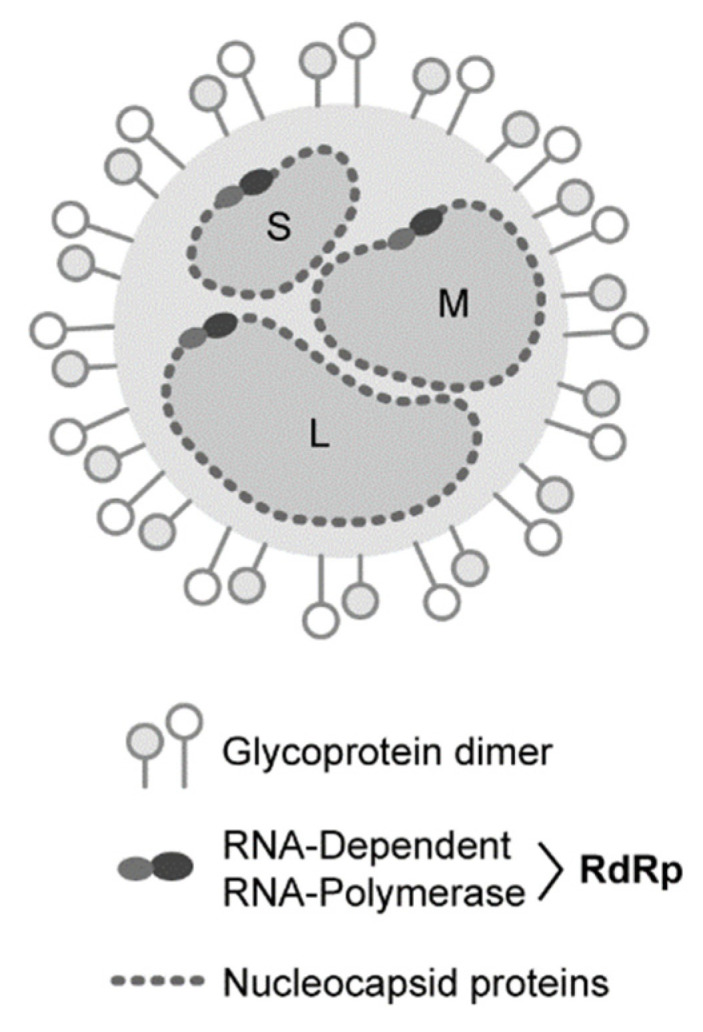
*Bunyavirales* Generalized Virion. Although bunyaviruses can vary in number of genomic segments and glycoprotein dimer structure, all viruses share a similar structure. Nucleocapsid proteins coat the single-stranded anti-sense RNA genomes and along with the RNA-dependent RNA-polymerase form ribonucleoproteins on each segment. The genomic segments are packaged in an enveloped virion studded with the viral glycoproteins. The genomic small, medium, and large segments are labeled S, M, and L, respectively.

**Figure 3:**
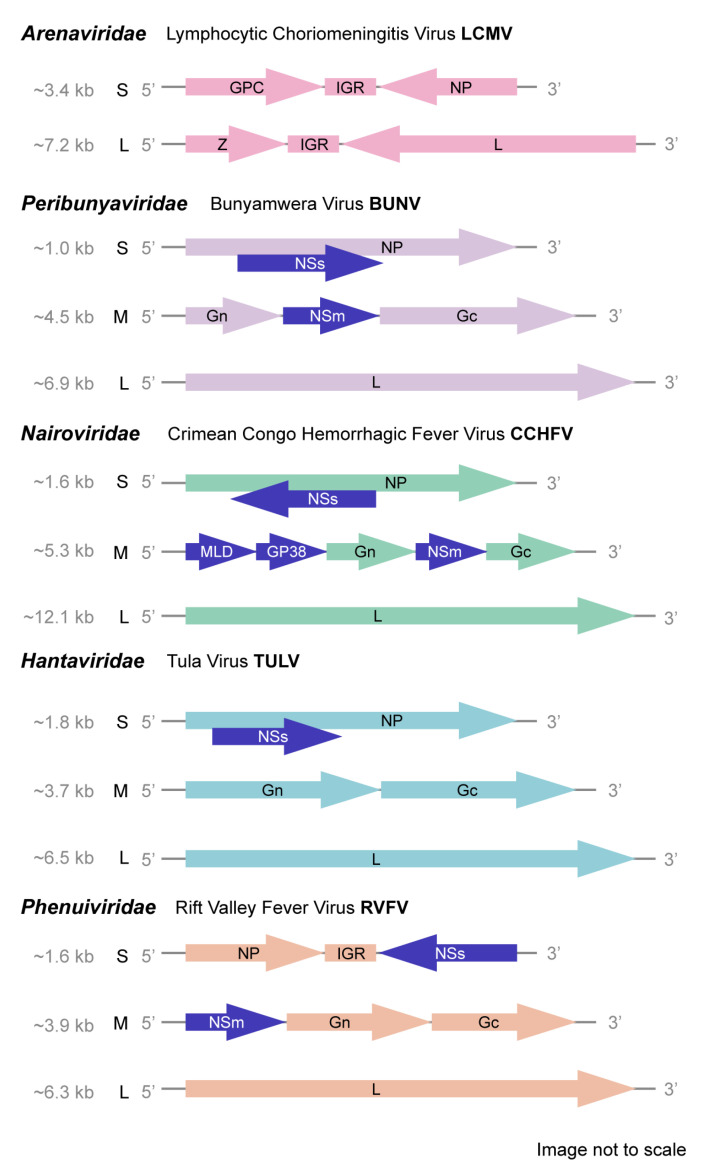
*Bunyavirales* Genome Organization. Specific genome structures for *Bunyavirales* families containing human pathogens, including *Arenaviridae*, *Peribunyaviridae*, *Nairoviridae*, *Hantaviridae*, and *Phenuiviridae* [5,12,13,14,15,16,17,18,19], are shown. The reference strain for each family is listed as well as the approximate size of each genomic segment. Glycoprotein precursor (GPC), nucleoprotein (NP), intergenic region (IGR), zinc-binding matrix protein (Z), non-structural S segment protein (NSs), non-structural M segment protein (NSm), surface glycoproteins (Gn and Gc), mucin-like domain (MLD), and secreted glycoprotein (GP38). NS proteins are highlighted in dark blue and include NSs and NSm, along with MLD and GP38 for *Nairoviridae*. The L segment is not as well understood as S and M and has many regions with unknown function but, across the order encodes the RNA-dependent RNA-polymerase (RdRp) gene.

Although these human pathogenic viruses are diverse in host pathogenicity, they share common features including segmented, linear, single-stranded antisense or ambisense RNA genomes [2]. Further, they are primarily transmitted by arthropod vectors including mosquitoes, ticks, and sandflies [3,4], although some such as arenaviruses and hantaviruses are spread directly from rodents to humans [5]. Bunyaviruses present a serious threat to public health as multiple viruses within the order are at risk of spread to non-endemic areas due to the expanding range of their vectors [4]. Further, these emerging viruses pose a continuous threat not only to human health but also to agriculture and livestock due to the diversity in targeted hosts [4]. Viruses of the *Bunyavirales* order share a similar structure. Multiple copies of the nucleocapsid (NP) protein encapsulate genomes, while the glycoproteins coat the enveloped virion [4,6] (Figure 2). The RNA-dependent RNA-polymerase (RdRp), along with the NPs, forms a ribonucleoprotein (RNP) on each genomic segment [4,6] (Figure 2), although some encode for additional functions. Across the order, genomes consist of two to six segments and this can vary even within specific families. For example, viruses in the *Arenaviridae*, *Wupedeviridae*, and *Unassigned* families typically have bi-segmented genomes, while viruses in *Fimoviridae* and the *Phenuiviridae* subfamily *tenuivirus* can have four to six [6,7,8,9,10,11]. However, generally bunyaviruses have three genomic segments: the small (S) segment of ~1–2kb, medium (M) segment of ~3.7–5kb, and the large (L) segment varying from ~6.8–12kb [12,13] (Figure 2 and Figure 3).

In addition to these essential proteins, many viruses within the *Bunyavirales* order encode non-structural (NS) proteins within the S and M segment. While the structural proteins have been well characterized across viral families, the NS proteins are not as well understood. As will be discussed in this review, the NS proteins of human pathogenic *Bunyavirales* are often key virulence factors and may be promising targets for effective interventions against infection. Of note, *Arenaviridae*, containing important human pathogens such as Lassa fever virus, Junin virus, and Lymphocytic Choriomeningitis virus, have only two genomic segments, the S and L, with no apparent NS proteins but an ambisense coding strategy [5]. However, the remaining families with human pathogens, *Peribunyaviridae*, *Nairoviridae*, *Phenuiviridae*, and *Hantaviridae*, all have similar tri-segmented genomes (Figure 3), and all encode NS proteins.

Interestingly, most studies have shown these NS proteins to interfere with host innate immune responses, suggesting common evolutionary pressures retained these proteins during divergence of the *Bunyavirales* order from its most recent common ancestor. In addition, in NSs, NSm, or double NSs/NSm mutants, viruses can become highly attenuated and/or less infectious, indicating important roles for these proteins in virulence. Throughout the order, antagonization of the host interferon response is the most ascribed function for NSs, while the roles of NSm vary but most commonly are associated with viral infection and replication. However, the families harbor rich diversity, and there is still much to uncover in the wide range of *Bunyavirales* NS protein function. This review will focus on the four aforementioned families which harbor human pathogens. As research is ongoing for therapeutics relevant to viruses in these families, understanding the current advances in NS protein role in virulence may be beneficial to intervention development.

## 2. Family Peribunyaviridae

The *Peribunyaviridae* family has 4 Genera—*Orthobunyavirus*, *Herbesvirus*, *Pacuvirus*, and *Shangavirus* [2]. The *Orthobunyavirus* genus is the most studied and understood of the family and includes viruses such as California Encephalitis virus (CEV), La Crosse Encephalitis virus (LACV), Bunyamwera virus (BUNV), Ngari virus (NRIV), and Batai virus (BATV). These viruses cause symptoms that range from mild febrile illness to encephalitis in humans and abortions in ruminants [20]. With mosquito vectors, these viruses have become increasingly concerning as a global health threat due to changing environmental conditions caused by climate change supporting vector spread [20]. The remaining genera are not known to cause illness in humans, and some do not encode NSs or NSm. *Herbesvirus* is not known to have any non-structural proteins, while both *Pacuvirus* and *Shangavirus* encode NSm, but not NSs. [21,22,23].

### 2.1. Functions of the NSs

A second open reading frame (ORF) exists in the S segment through a +1 frameshift of various viruses in the *Peribunyaviridae* family, resulting in the production of the NSs protein (Figure 3) [24]. Early research into the truncated protein showed that its size was approximately 10 kDa and, depending on the C-terminus, could range from 89–103 amino acids [25]. It appears that the majority of *Orthobunyavirus* members do not contain the NSs ORF [26], and there may be an evolutionary trend towards encoding smaller NSs proteins, possibly due to fewer codons reducing chances of detrimental mutations [27]. The main role of NSs in *orthobunyaviruses* appears to be antagonizing the host immune response, primarily though blocking the production of type I IFN [24]. Expression of BUNV NSs in vitro was found to block double-stranded RNA (dsRNA)-mediated induction of IFN-β [28]. IFN induction by dsRNA is mediated by melanoma differentiation-association gene 5 (MDA5) and retinoic acid inducible gene 1 (RIG-I) [29]. MDA5 and RIG-I are activated by the presence of dsRNA and signal through mitochondrial antiviral signaling protein (MAVS) to activate interferon response factors 3 and 7 (IRF3/7) leading to production of Type 1 IFN [30,31]. NSs does not affect IRF3 activation but rather acts downstream of this signaling event as IRF3 dimerization was not impacted by LACV NSs expression, indicating that inhibition must occur at the cellular RNA transcription level [29]. In BUNV, NSs blocks the phosphorylation of the major subunit of RNA Polymerase II, thereby blocking transcription [28]. This correlates with nuclear and cytoplasmic localization of the BUNV NSs [32]. LACV NSs deletion mutants show strong induction of IFN-β, suggesting that this is the primary mechanism of IFN inhibition for some *Orthobunyaviruses* [29]. BUNV NSs also inhibits RNA polymerase phosphorylation through similar mechanisms to LACV and interacts with mediator protein MED8 to antagonize the innate immune response [33]. These data were obtained using minigenome systems and further in vitro and in vivo studies are warranted.

Further, some *Orthobunyaviruses* possess alternative methods for blocking type I IFN. Despite not having an NSs protein, Tacauima virus (TCMV), inhibits type I IFN [27,34] although the mechanism by which TCMV achieves this is unknown, highlighting an area for further research. Interestingly, Umbre virus and Witwatersrand virus, which both encode an NSs, do not cause illness in humans indicating that NSs alone is not sufficient for human virulence [26]. In addition, NSs has been linked to apoptosis in virally infected cells [24]. In CEV and LACV, the NSs protein was found to have a sequence similar to Reaper, a pro-apoptotic protein found in *Drosophila* [35]. Reaper is an inhibitor of inhibitor of apoptosis (IAP) molecules and serves to promote caspase mediated apoptosis and mitochondrial cytochrome C release [35,36]. Cells infected with rLACV with and without NSs were monitored for DNA breaks, a key indicator of apoptosis, and it was found that fragmentation occurs in cells infected with rLACV possessing NSs but no fragmentation could be detected in cells infected with rLACV lacking NSs [37]. In contrast to the pro-apoptotic function of the LACV NSs, BUNV NSs has been shown to strongly inhibit apoptosis which may facilitate efficient replication [37,38]. Specifically, BUNV NSs inhibits IRF3-mediated apoptosis by suppressing an IRF3 dependent promoter which is induced in the cell following BUNV infection [38]. A recombinant BUNV lacking NSs induced apoptotic cell death more rapidly than wild-type virus and supports the role of BUNV NSs in this pathway [38]. Further, the BUNV NSs was also shown to inhibit the induction of IFN α/β to suppress antiviral signaling [38].

### 2.2. Potential Roles of the NSm

Compared to the NSs, little is known about the role that NSm plays in *Orthobunyaviruses*. A single ORF encodes the M segment polyprotein which later is cleaved into Gn, Gc, and NSm proteins, with the NSm located in between the glycoproteins (Figure 3) [24,39]. Approximately 16–18 kDa, NSm contains three hydrophobic and two hydrophilic domains [24,40], although there is still debate as to how the NSm is cleaved from the glycoprotein precursor (GPC). Within the NSm itself, it appears that not all domains are required, as deletion or mutation of the C terminus does not impair BUNV ability to infect mammalian cells [40,41]. However, BUNV experiments show that NSm may play a role in infection as a scaffolding protein and accumulates near the Golgi apparatus [42]. NSm localizes to the cylindrical and globular domains of the viral tubes made during infection and replication, and the lack of NSm in mutant BUNV led to less stable tubular structures and immature viral particle accumulation, pointing to a potential role in viral assembly [42]. In vivo studies involving mutant Akabane virus with NSm partially deleted have shown significant reduction in plaque size and reduction in pathogenicity, although neuro-invasiveness and neurovirulence were retained, indicating a minor potential role in infection [43].

## 3. Family Nairoviridae

The *Nairoviridae* family includes known human pathogens such as Crimean-Congo Hemorrhagic Fever virus (CCHFV) [44] and non-human pathogenic viruses including Dugbe virus (DUGV) [45], Nairobi Sheep Disease virus (NSDV) [46], and Hazara virus (HAZV) [47]. These viruses share common characteristics in both environmental transmittance and genomic organization. While hosts range between birds, humans, rodents, and ruminants, among others, they are most commonly maintained in and transmitted by arthropods such as ticks [12]. *Nairoviridae*, compared to other bunyaviruses, have a more complex genomic M segment and larger L [48] (Figure 3). Interestingly, these viruses are most closely related to the *Bunyavirales* family *Wupedeviridae* (Figure 1), which, as of July 2019, contains a single virus: Wuhan millipede virus 2 [2,12]. In respect to the NS proteins, most research is aimed at CCHFV. Endemic in most of Asia, Africa, and Europe, CCHFV infection, true to its name, causes hemorrhagic fevers with a fatality rate of up to 30% [12,48]. With currently no vaccines or therapeutics [49,50], further study is needed to understand CCHFV pathogenesis and produce an efficacious intervention. Among efforts is research seeking to elucidate the role of NS proteins in disease.

### 3.1. Potential Roles of the NSs

The CCHFV NSs is the most well-studied of the *Nairoviridae* NSs proteins and is encoded in an opposite sense ORF of the genomic S segment (Figure 3). It contains 150 amino acid residues, and is highly conserved amongst CCHFV isolates [50,51], indicating a potential critical function retained over viral divergence. Interestingly, multiple CCHFV susceptible cell lines, including VeroE6, Hela, and 293FT cells, show that NSs is vulnerable to degradation by proteasomes but over-expression induces apoptosis [50]. In these cell lines, inducing overexpression of NSs resulted in significantly higher levels of caspase-3/7 activity, indicating activation of the apoptosis extrinsic pathway [50]. Interestingly, this was also found to be true in human SW13 cells infected with the closely related HAZV virus [52]. Both CCHFV and HAZV induced apoptosis at 48hrs post-infection through this pathway [50,52]. In addition, NSs disrupts mitochondrial membrane potential and thus also activates apoptosis through the intrinsic pathway [50]. CCHFV interaction with the host cell apoptosis machinery may be key to viral replication, as CCHFV possesses proteins with both pro- and anti-apoptotic function, possibly to regulate host cell health in sync with viral replication [53,54]. Thus, host cell degradation of NSs may be a defense mechanism, although further studies are needed to describe how exactly NSs regulates apoptosis and supports viral replication [50]. To date, CCHFV NSs has no described role in antagonizing the host interferon response, as has been described for the NSs of other *Bunyavirales* discussed in this review. Interestingly, mouse-adaptation of CCHFV to immunocompetent mice resulted in a mutation to the CCHFV NSs suggesting NSs may play a role during pathogenesis in vivo [55].

### 3.2. Nairovirus-Specific NS Proteins, Mucin-Like Domain (MLD) and GP38

Comparative analysis with predictive software of the deduced amino acid sequences of 14 South African CCHFV isolates shows highly conserved proteolytic cleavage sites along the genomic M segment which result in generation of two structural glycoproteins Gn and Gc, and three non-structural proteins—NSm, the secreted mucin-like domain (MLD), and GP38 (Figure 3) [48]. The MLD is highly divergent among isolates, possessing little conservation at either the nucleotide or amino acid level [48,56]. Although the function of the MLD in CCHFV pathogenesis is unknown, Ebola virus (EBOV), order *Mononegavirales* [57], has an MLD which disrupts endothelial cells [58]. However, the EBOV MLD is a domain of the structural glycoprotein [58] and likely unique from the CCHFV MLD [48]. In a study of CCHFV transcriptionally competent virus-like particle (tc-VLP) replication, deletion of MLD had no impact on particle infectivity although it reduced incorporation of glycoproteins into particles by about 60%, while the MLD-GP38 double deletion inhibited assembly of infectious tc-VLPs [59]. GP38, encoded between the MLD and pre-Gn of the M polyprotein (Figure 3), is generated by host proteases using highly conserved furin and SKI-1 cleavage sites [48]. However, CCHFV mutants lacking this furin site, and thereby lacking optimal GP38 release, have only slightly decreased Gn maturation and transient reduction in virus titers, indicating that the furin site is not required for viral replication, and the reduction in viral titers may be due to either or both of decreased GP38 and mature Gn production [44]. In the same tc-VLP experiment, loss of infectivity from MLD-GP38 double deletion was associated with impaired Gc maturation, showing a dual effect of GP38 on both Gn and Gc trafficking to the Golgi, where both proteins are processed [44,59]. This indicates that the MLD may have conformational effects on GP38 that impact its ability to traffic Gc to the Golgi or, MLD may regulate Gc accumulation in the Golgi [59]. Interestingly, targeting of the GP38 by host antibody responses may be protective as mice treated with a non-neutralizing monoclonal antibody recognizing GP38 were protected against lethal disease [56,60]. Sequence analysis of viruses in the CCHFV and NSDV serogroups which include HAZV and DUGV, respectively, suggests a similar presence of GP38, and thus these viruses may benefit from similar antibody therapeutics [56].

### 3.3. Viral Assembly and Infectivity Impacted by the NSm

The double membrane spanning NSm was identified in 2007 and is found in CCHFV, DUGV, NSDV, and HAZV [47,59,61]. NSm is released in the endoplasmic reticulum (ER) where, for CCHFV, the glycoprotein precursor encoded by the genomic M segment is cleaved by subtilase-like proteases into NSm, preGn, and preGc (Figure 3) [62]. In tc-VLP experiments, NSm deletion caused improper Gc processing, defective particle formation, and impaired secretion, although initial trafficking of Gc was unaffected [48,59]. Interestingly, another study, using recombinant CCHFV with NSm deletion, observed that NSm is not essential for viral replication in vitro, as viral growth was only mildly slower compared to WT virus [18]. Further, interferon alpha receptor deficient mice (IFNAR^−/−^) mice infected with rCCHFV lacking NSm succumbed to severe and lethal disease, albeit at a delay [18], demonstrating NSm is not essential for lethal disease in vivo, at least in the absence of type I IFN. However, mouse adaptation of CCHFV to immunocompetent mice resulted in a mutation in NSm, suggesting NSm may play a role in type I IFN competent hosts [55]. These differences may be attributable to the function of NSm combatting Golgi retention or a yet to be characterized function. DUGV and NSDV, which occasionally infect humans [45,46], also encode for NSm. In HAZV, which does not infect humans, the NSm has a 43 amino acid deletion in the cytoplasmic domain [47], possibly suggesting a role for NSm in human virulence. However, another *Nairoviridae* member, Erve virus, lacks any NSm sequence and has been shown to cause disease characterized by “thunderclap headaches” and intracerebral hemorrhage in humans [63]. Thus, further research is needed to fully elucidate the role of NSm in both CCHFV and *Nairoviridae* disease.

## 4. Family Hantaviridae

Newly reclassified as a viral family in 2016 [16], *Hantaviridae* contains several viral subfamilies and different genera. Viruses in the genus *Orthohantavirus* are historically classified as Old and New World based on the global distribution of their primary rodent reservoir and the corresponding clinical syndrome they cause [16,64]. Old world viruses such as Hantaan and Dobrava-Belgrade orthohantavirus are known to cause hemorrhagic fever with renal syndrome (HFRS) mainly in Asia and Eastern Europe [17]. Seoul orthohantavirus has the potential to cause disease worldwide due to the global distribution of its reservoir *Rattus rattus* or *Rattus norvegicus*, and Puumala orthohantavirus, harbored across Central Europe and Scandinavia in *Myodes glareolus*, is the causative agent of a mild HFRS designated nephropathia epidemica [65]. New World viruses such as Sin Nombre (SNV) and Andes orthohantavirus (ANDV), carried in *Peromyscus maniculatus* and *Oligoryzomys longicaudatus*, respectively, are the main causes of hantavirus (cardio) pulmonary syndrome (HPS or HCPS) in the Americas [17]. Common transmission between rodents occurs through bites or aerosolized rodent saliva, urine, and feces, and although these rodents can be chronically infected, they are not affected otherwise by hantaviruses. Transmission from rodent to human is also through aerosolized rodent saliva, urine, and feces [17]. The *Hantaviridae* genome encodes an NSs on the S segment, although no NSm has been identified (Figure 3) [17]. In comparison to other *Bunyavirales*, the NSs of the *Hantaviridae* is not well understood and not present in all members of the family.

### Functions of the NSs

The NSs of the *Hantaviridae* range from 7–10 kDa, or approximately 90 amino acids [65,66]. The protein is translated from a +1 frameshift ORF or leaky scanning, and only a few of the *Orthohantavirus* encode for an NSs including ANDV, Puumala virus (PUUV), and Tula Virus (TULV) [66], although it is believed that other viruses including SNV encode NSs that are yet to be identified [66,67]. ANDV NSs was only recently discovered after analysis of viral small mRNA in the genome found a second initiation site, producing NSs of 63 amino acids similar to the putative SNV NSs domain of the same size, whereas PUUV and TULV NSs proteins are about 90 amino acids [67]. While the TULV and PUUV NSs demonstrate the ability to block IFN and NF-kB signaling [68,69], it is unknown how ANDV NSs functions in infected cells, although current evidence suggests it works in a similar fashion to other *Hantaviridae*. Compared to other *Bunyavirales*, NSs in orthohantaviruses appear to be less effective at inhibiting IFN and other aspects of the innate immune response [68,69]. This may be a factor in how orthohantaviruses establish persistent chronic infections. By limiting dsRNA production during infection, these viruses can regulate a modest induction of the IFN pathway [69]. There has also been a concerted effort to determine the cellular partners for *Hantaviridae* NSs to better understand their role during viral infection. Data analysis for PUUV and TULV NSs found evidence for interactions with host proteins Keratin 14 (KRT14), Actin-Related Protein 5 (ACTR5), and Acyl-coenzyme A binding domain containing 3 (ACBD3) [70]. ACTR5, interestingly, is involved in chromatin remodeling, reminiscent of the *Phenuiviridae* Rift Valley fever virus (RVFV) NSs, discussed below. ACBD3 is known for its role in maintaining Golgi structure and may recruit factors needed for viral replication. Other viral NSs, such as the ones found in order *Picornavirales*, are known to interact with ACBD3 for manipulating the host immune response [70]. Overall, further research is needed to fully understand the role of *Hantaviridae* NSs in infection and disease progression.

## 5. Family Phenuiviridae

The *Phenuiviridae* family includes animal, plant, insect, and human pathogens [11,19]. Human pathogens are generally transmitted by ticks or phlebotomus sandflies, although RVFV, a notable human pathogen, is an outlier transmitted by *Aedes* and *Culex* mosquitos [19]. These viruses cause a wide range of symptoms in both humans and animals from mild febrile illness to meningitis and hemorrhagic fever in humans, or, for example, hepatitis, hemorrhage, and abortion in cattle and sheep infected with RVFV [19]. Research has primarily focused on characterizing disease in livestock and humans since these pose the biggest threat to the population and agricultural economy [19]. However, the vector species are important for viral maintenance and transmission as well as geographic distribution [19]. RVFV and other *Phenuiviridae* human pathogens, including Severe fever with thrombocytopenia syndrome virus (SFTSV), Heartland virus (HRTV), Punta Toro virus (PTV), and Toscana virus (TOSV) share a similar genomic organization of structural and NS proteins. While the genomic L and M segments are of negative polarity [11], the S segment is uniquely ambisense, encoding the NP and an antigenomic NSs (Figure 3) [19]. Interestingly, while the NSs is weakly conserved across *Phenuiviridae* in amino acid sequence, its function as an interferon (IFN) antagonist is highly conserved [19]. The NSs protein of the *Phenuiviridae* members RVFV and SFTSV are among the most well studied NS proteins of the *Bunyavirales* order. The NSm, preceding the Gn and Gc in the M segment ORF (Figure 3), is not as extensively studied but is thought to play a role in maintaining infection in viral vectors [11].

### 5.1. Overview of RVFV NSs Roles in Virulence and Vaccine Development

RVFV primarily infects ruminants and causes significant damage to livestock, but can also be fatal in humans [71]. To date, RVFV NSs has been extensively studied as a major virulence factor and is known to have several functions aiding in viral evasion of host immunity and increased pathogenicity including inhibition of general transcription, degradation of protein kinase R (PKR), segregation of chromatin DNA, nuclear accumulation and filament formation, apoptosis activation, and antagonism of type I IFN system [72,73,74]. RVFV NSs induces cellular damage through various mechanisms including interactions with mitochondria [75], proteasome [76], SMAD proteins [77], nuclear pore protein Nup98 [78], casein kinase II [79], p62 involved in general transcription [80], p53 involved in cell cycle and apoptosis regulation [81], and ABl2 and the actin cytoskeleton [82]. In RVFV NSs coding region mutants, virulence is decreased and infection can be characterized by lack of filament formation in nuclei of infected cells [83], decreased IFN antagonism and inability to degrade PKR [84], reduced general transcription inhibition and cytotoxicity [80], and reduced ability to inhibit antiviral signaling by macrophages [85]. Additionally, human host cell protein STAT3 (signal transducer and activator of transcription 3), a pro-survival protein, specifically targets RVFV NSs to inhibit apoptosis and influence NSs nuclear localization [86]. In mosquito cells, RVFV NSs is expressed at significantly lower levels [87,88]. Cells of known mosquito hosts showed distinct pathways via dicer-2 and piwi-mediated RNA interference that suppress NSs filament formation and allow for antiviral responses against secondary RVFV infection [89]. Cumulatively, NSs interacts with multiple host pathways to promote viral replication, while distinct immune responses against NSs in insect vectors may promote vector competence for RVFV.

Interestingly, in analyses of naturally infected animals, studies show that NSs is not a major target of the host adaptive response [90], and since NSs is a significant virulence factor for RVFV, many studies have utilized RVFV NSs mutants for vaccine development. Natural RVFV isolate clone 13 contains a 70% NSs deletion, is avirulent and highly immunogenic in mice and hamsters [91,92], and protective as a pre-exposure vaccine against viremia and clinical symptoms in lambs [93]. Another strain, MP-12 containing the clone 13 NSs deletion, protected hamsters by post-exposure vaccination, while the parental MP-12 strain did not, indicating that successful inactivation of NSs was important for vaccine efficacy [94]. The parental MP-12 strain, which originated as a mutant from 12 serial passages of the natural virulent RVFV isolate ZH548, encodes a functional NSs protein with a single amino acid change [95] and a variety of further studies support deletions or increased mutation of NSs to improve MP-12 vaccine efficacy [96,97,98,99]. In respect to safety, these mutants are able to replicate efficiently but are unable to shut off host protein synthesis in vitro [100]. Additionally, live attenuated vaccines, distinct from MP-12, achieved protective efficacy with NSs deletions [101,102,103,104] as did antiviral treatments such as bortezomib [105] and curcumin [106] which target NSs or interfere with NSs-host protein interactions. Overall, targeting of NSs in successful vaccines and antivirals indicate the importance of this NS protein in disease virulence and the need to account for it in intervention development.

### 5.2. The NSs Across Phenuiviridae

Within a group of 18 RVFV strains, NSs varied by 0 to 9.5% at the amino acid level [107] while the NSs sequences of Sandfly Fever Sicilian Virus (SFSV), PTV, TOSV, and Uukuniemi virus (UUKV) (listed in increasing divergence from RVFV) differ by 17–30% [108]. Interestingly, while RVFV NSs proteins localize in the nucleus, those of SFTSV, TOSV, and UUKV localize in the cytoplasm [72]. UUKV, which is otherwise closely related to RVFV, is not recognized as a human pathogen. UUKV NSs, a weak interferon antagonist in human cells [109], is only known to associate with the 40s ribosomal subunit [110] and interact with MAVS [111]. For Arumowot virus (AMTV), another non-human pathogen, the NSs is rapidly degraded via proteasome [112]. Considering viruses such as UUKV and AMTV that have low NSs–host protein interactions and severe disease causing pathogens such as RVFV that have multiple interactions, the efficiency of the virus NSs–host interaction may be correlated to disease severity [111]. PTV and SFSV, which have NSs ~85% divergent from UUKV [113], are pathogenic in humans and have characterized functions similar to RVFV. PTV NSs, such as RVFV, and reminiscent of the *Nairoviridae* CCHFV NSs, induces apoptosis both extrinsically and intrinsically through activation of caspase-3, -8, and -9 [114]. PTV NSs also has a type I IFN antagonist function, inhibiting IFN-α/β, although the extent of this varies across PTV variants that diverge in the NSs gene specifically [115]. Interestingly, although the PTV NSs and RVFV NSs share interferon antagonist functions, they have very little homology, sharing only 27 common residues out of 250 [115]. Further, SFSV, which causes a spectrum of transient febrile illness to severe neuro-invasive disease [116], has an NSs which suppresses the type I IFN system by interfering with tank-binding kinase 1 (TBK1)-IRF3 association with the IFN-β promoter [117]. This direct masking of IRF3 DNA-binding domain is unique to less virulent human pathogenic *Phenuiviridae*, as the highly virulent tend to completely destroy or sequester host factors [117]. TOSV NSs, which causes central nervous system (CNS) infections [118], also exhibits functions similar to that of RVFV and PTV including IFN-β antagonism via RIG-1 degradation and IRF3 inhibition [119,120] although this function is weaker than that seen in other *Bunyavirales* [121]. Additionally, TOSV NSs associates with viral nucleocapsids in mature virions, indicating a potential function in viral replication [122]. In TOSV NSs full deletion or C-terminal deletion mutants, TOSV loses the ability to suppress IFN-β [123,124] and, interestingly, replacement of SFSV NSs C-terminal domain with that of TOSV confers the ability to SFSV to degrade RIG-1 via ubiquitination [124]. Studies characterizing a combination RVFV MP-12 vaccine with TOSV NSs showed loss of hepatic disease while retaining neuro-invasiveness in mice, indicating differences between the RVFV and TOSV NSs requiring further study [125]. Lastly, TOSV, unlike PTV and SFSV, downregulates PKR but does not affect cellular transcription during infection, whereas RVFV affects both [126].

Further, HRTV, SFTSV, and Guertu virus (GTV) are genetically closely related *Phenuiviridae* with functional NSs proteins [127]. HRTV and SFTSV, both discovered in 2009 [128,129], have similar NSs that function to suppress the type I IFN system and, uniquely, the type II (SFTSV NSs only [130]) and type III IFN systems [131,132]. Mainly, SFTSV and HRTV NSs suppress the phosphorylation of TBK1/IKKε-IRF3 signaling pathway as a means of inhibiting IFN-β production [111,133,134,135]. This contrasts with RVFV NSs which doesn’t interact with TBK1 directly in the cytoplasm but instead suppresses its expression levels and ability to activate IRF3, thus resulting in similar IFN-β inhibition [134]. SFTSV or HRTV NSs and TBK1 association, similar to UUKV, suppresses MAVS-mediated activation of IFN-β expression [135]. Further, SFTSV NSs interferes with the type I IFN system via formation of inclusion bodies (IBs). Specifically, SFTSV NSs is implicated in suppressing IFN-β via re-localization of RIG-I, TRIM25, and STAT2 into these IBs [136,137,138,139]. Sequestering of STAT2 inhibits the JAK/STAT pathway and decreases interferon-sensitivity response element (ISRE) activity [140]. IB formation by SFTSV involves localization of NSs with perilipin A and ADRP which are present in the cellular host lipid droplets utilized by the virus as the basis of the IBs [141]. After this localization, translocation by NSs of synaptogyrin-2 into formed IBs promotes a stable environment for viral replication [141]. Interestingly, GTV, discovered in 2018 [142], also inhibits type I IFN signaling by inducing formation of IBs and extended filamentous structures (FSs) where, via interactions with IRF3 and STAT2, it sequesters host proteins [127]. While serological antibody surveys suggest that GTV can infect humans, additional research is needed to fully understand potential threat as a human pathogen [142]. HRTV NSs has not been shown to utilize IBs but does interact with STAT2 and impairs IFN-β induced phosphorylation in the cytoplasm [111,133]. Further, SFTSV and HRTV inhibit IFN-α [132]. SFTSV NSs reduces IFN-α by trapping IRF7 in IBs, blocking IRF7′s normal functions in innate immunity and induction of IFN-α expression [143]. HRTV NSs is thought to affect IFN-α by interactions with STAT2 [132]. Additionally, unlike any of the previous viruses discussed, SFTSV NSs has been shown to interfere with type II IFN signaling [130]. Specifically, the NSs protein either downregulates STAT1 protein abundance or sequesters STAT1 in viral inclusion bodies (IBs) to block IFN-γ receptor signaling [130]. Further, SFTSV/HRTV NSs antagonizes type III IFN through inhibition of IFN-λ and blocking phosphorylation and nuclear transport of STAT1 and STAT2, although in the case of HRTV, a mechanism independent of NSs is thought to be responsible for STAT 1 interactions [131,132].

Lastly, SFTSV NSs functions to increase virulence independent of the IFN systems. This includes upregulating the p62-Keap1-Nrf2 antioxidant pathway [144], inducing interleukin 10 (IL-10) expression involved in host immune response [145], suppressing NF-κB promoter activities to avoid innate immunity signaling [146], interacting with TRIM25 to mediate antiviral signaling [147], and promoting cell cycle arrest [148]. Further, although SFTSV NSs is dispensable for viral replication [128], it can form viroplasm-like structures (VLSs) in infected cells and these serve as sites of viral dsRNA localization, indicating a potential novel role of NSs in enhancing SFTSV replication [149]. In infected Vero cells, SFTSV NSs localizes with autophagy pathway proteins LC3B, p62, and Lamp2b indicating a potential virulence characteristic of SFTSV to hijack cellular autophagy processes and increase pathogenesis [137].

### 5.3. Potential Roles of the NSm

The NSm protein, preceding the Gn and Gc in the M segment ORF (Figure 3) [11], is not as extensively studied as the NSs and is not present in all *Phenuiviridae* as SFTSV, HRTV, and GTV lack an NSm. For RVFV, cleavage of the GPC encoded by the M segment results in either of two forms of NSm—the general 14kDa cytosolic protein or the NSm-Gn (NSm’) 78kDa glycoprotein [150]. RVFV NSm mutants result in inhibition of viral infection and dissemination in both *Culex quinquefasciatus* and *Aedes aegypti* mosquitoes [150,151], the major vectors of the virus [71] and where NSm expression is normally upregulated [88]. Double NSm/NSm’ RVFV deletion mutants are highly attenuated in tissue culture and in mice [150]. Further, in host cells, RVFV NSm tends to accumulate near the nucleus, as opposed to accumulation in the cytoplasm in mosquito cells [88]. Interestingly, RVFV NSm was the first identified protein of the family to have an antiapoptotic function in host cells [152]. The protein, dispensable for viral replication [152,153,154], suppresses caspase -3, -8, and -9 activation [152], integrates into the mitochondrial outer membrane [155], and regulates cellular p38 MAPK [156]. Further, screening of a mouse cDNA library revealed putative cellular protein interactions with RVFV NSm including SNAP-25, Ppil2, and Cpsf2 which indicate potential roles of NSm in neuro-invasion, protein trafficking, and mRNA nuclear transport, respectively [157]. However, RVFV NSm deletion mutants are uniformly lethal in rats, indicating that NSm is not essential for virulence [101]. Lastly, comparing the M segment across *Phenuiviridae*, PTV has high M segment homology to RVFV except for its putative NSm region which is much larger [158]. Inducing expression of this region resulted in viral protein retainment in the ER and inability to traffic to the Golgi for proper processing [159]. As PTV causes milder illness than RVFV, further research is needed to uncover if this is related to the divergent NSm region. Further, TOSV was verified to have an NSm in the same location of RVFV [160], but the amino acid sequence is 62% divergent from RVFV with little similarity to PTV and UUKV NSm regions [161]. Overall, further research is needed across the *Phenuiviridae* family to characterize the role of NSm in virulence.

## 6. Conclusions

Considering the high degree of diversity throughout the order *Bunyavirales* and its 13 families, it is interesting to note the human pathogen-containing families have conserved NS protein functions, including interferon antagonism (NSs), interference with host cell apoptosis (NSs), supporting viral replication (NSs/NSm), and facilitating infection (NSm) (Table 2). These common functions persist despite the significant variation in how these NS proteins are encoded in viral genomes (Figure 3). While the *Phenuiviridae* RVFV NSs is the most extensively studied NS protein of those discussed in this review, similarities in protein function have been found in other members of the *Phenuiviridae* family and among *Peribunyaviridae, Hantaviridae*, and *Nairoviridae* viruses (Table 2), showing that conserved functions of these NS proteins may be critical for human pathogenicity.

However, further research is needed to fully determine the role of NS proteins in viral pathogenesis across the *Bunyavirales* order. The significant attenuation of many viruses engineered to lack an NSs and the diversity in interactions between viral NSs and host proteins present a variety of therapeutic strategies and targets to limit severe disease caused by these viruses. NSm, although divergent across *Bunyavirales*, has some conserved function in viral infectivity and interference in host immunity. As the NSm is not as well understood as NSs, further research may show new and exciting functions, lending to overall *Bunyavirales* knowledge and informing development of future interventions. Cumulatively, despite wide diversity in geographic range, susceptible hosts, vector species, human disease, and genomic organization, the general conservation of NS proteins across the *Bunyavirales* order indicates these are important viral proteins that may mediate the severity of human morbidity and mortality caused by these viruses.

## Figures and Tables

**Table 1:** Order *Bunyavirales* Taxonomy. Taxonomical classification of the 13 *Bunyavirales* families according to the most recent International Committee on Taxonomy of Viruses (ICTV) taxonomy update. Included are identified type species for each family as well as relevant viruses discussed in this review [2].

Family	Genus	Species	Common Name
*Mypoviridae*	*Hubavirus*	* Myriapod hubavirus	Húběi Myriapoda Virus 5 (HbMV-5)
*Wupedeviridae*	*Wumivirus*	* Millipede wumivirus	Wǔhàn Millipede Virus 2 (WhMV-2)
*Nairoviridae*	*Orthonairovirus*	* Dugbe orthonairovirus	Dugbe Virus (DUGV)
		*Crimean-Congo hemorrhagic fever orthonairovirus*	Crimean-Congo Hemorrhagic Fever Virus (CCHFV)
		*Hazara orthonairovirus*	Hazara Virus (HAZV)
		*Nairobi sheep disease orthonairovirus*	Nairobi Sheep Disease Virus (NSDV)
		*Thiafora orthonairovirus*	Erve Virus (ERVEV)
*Tospoviridae*	*Orthotospovirus*	* Tomato spotted wilt tospovirus	Tomato Spotted Wilt Virus (TSWV)
*Peribunyaviridae*	*Orthobunyavirus*	* Bunyamwera orthobunyavirus	Bunyamwera Virus (BUNV)
			Ngari Virus (NRIV)
		*Akabane orthobunyavirus*	Akabane virus (AKAV)
		*Batai orthobunyavirus*	Batai Virus (BATV)
		*California encephalitis orthobunyavirus*	California Encephalitis Virus (CEV)
		*La Crosse orthobunyavirus*	La Crosse Virus (LACV)
		*Tacaiuma orthobunyavirus*	Tacaiuma Virus (TCMV)
		*Turlock orthobunyavirus*	Umbre Virus (UMBV)
		*Witwatersrand orthobunyavirus*	Witwatersrand Virus (WITV)
*Arenaviridae*	*Mammarenavirus*	* Lymphocytic choriomeningitis mammarenavirus	Lymphocytic Choriomeningitis Virus (LCMV)
		*Lassa mammarenavirus*	Lassa Virus (LASV)
		*Argentinian mammarenavirus*	Junin Virus (JUNV)
*Hantaviridae*	*Orthohantavirus*	* Hantaan orthohantavirus	Hantaan Virus (HTNV)
		*Dobrava-Belgrade orthohantavirus*	Dobrava Virus (DOBV)
		*Andes orthohantavirus*	Andes Virus (ANDV)
		*Puumala orthohantavirus*	Puumala Virus (PUUV)
		*Seoul orthohantavirus*	Seoul Virus (SEOV)
		*Sin Nombre ortohantavirus*	Sin Nombre Virus (SNV)
		*Tula orthohantavirus*	Tula Virus (TULV)
*Leishbuviridae*	*Shilevirus*	* Leptomonas shilevirus	Leptomonas Moramango Virus (LEPMV)
*Phenuiviridae*	*Banyangvirus*	* Huaiyangshan banyangvirus	Severe Fever with Thrombocytopenia Syndrome Virus (SFTSV)
		*Heartland banyangvirus*	Heartland Virus (HRTV)
		*Guertu banyangvirus*	Guertu Virus (GTV)
	*Phlebovirus*	* Rift Valley fever phlebovirus	Rift Valley Fever Virus (RVFV)
		*Punta Toro phlebovirus*	Punta Toro Virus (PTV)
		*Salehabad phlebovirus*	Arumowot Virus (AMTV)
		*Sandfly fever Naples phlebovirus*	Sandfly Fever Sicilian Virus (SFSV)
			Toscana Virus (TOSV)
		*Uukuniemi phlebovirus*	Uukuniemi Virus (UUKV)
*Unassigned*	*Coguvirus*	* Citrus coguvirus	Citrus Concave Gum-Associated Virus (CCGaV)
*Cruliviridae*	*Lincruvirus*	* Crustacean lincruvirus	Wēnlǐng Crustacean Virus 9 (WICV-9)
*Fimoviridae*	*Emaravirus*	* European mountain ash ringspotassociated emaravirus	European Mountain Ash Ringspot-Associated Virus (EMARaV)
*Phasmaviridae*	*Feravirus*	* Ferak feravirus	Ferak Virus (FRKV)

* Type species.

**Table 2:** Overview of NS Protein Functions. Overview of the various functions of non-structural (NS) proteins discussed for human pathogen-containing *Bunyavirales* families: *Peribunyaviridae*, *Nairoviridiae*, *Hantaviridae*, and *Phenuiviridae*.

Family	NS Protein	Functions
*Peribunyaviridae*	NSs	Blocking production of type I IFN [27,28,29,30,31,38]
		Blocking transcription and translation [28,29,162]
		Inducing apoptosis [24,162] Inhibiting apoptosis [38]
	NSm	Potential role in infection [42,43]
*Nairoviridae*	NSs	Inducing apoptosis [50,52]
	MLD	Potential role in Gn/Gc incorporation into viral particles [59]
		Potential impact on GP38 conformation [59]
		Potential role in regulating Gc accumulation in the Golgi [59]
	GP38	Potential role in Gn/Gc maturation [44,59]
		Potential role in viral replication [56]
	NSm	Role in viral replication and particle formation [48,59]
		Potential role in virulence [47]
*Hantaviridae*	NSs	Blocking IFN signaling [68]
		Blocking NF-kB signaling [69]
		Limiting dsRNA production [69]
		Potential role in chromatin remodeling [70]
		Potential role in viral replication [70]
*Phenuiviridae*	NSs	Aiding in viral evasion of host immunity [72,73,74]
		Inhibiting general transcription [72]
		Downregulating or Degrading PKR [72,126]
		Segregating chromatin DNA [72]
		Role in filament formation [72]
		Inducing apoptosis [72]
		Antagonizing of type I IFN system [72,111,115,117,119,120,127,132,133,134,135,136,137,138,139,140,143]
		Antagonizing of type II IFN system (SFTSV only) [130]
		Antagonizing of type III IFN system (SFTSV/HRTV only) [131,132]
		Inducing cellular damage [75,76,77,78,79,80,81,82,148]
		Inducing apoptosis [81,114]
		Promoting viral replication [122,149]
		Upregulating the p62-Keap1-Nrf2 antioxidant pathway [144]
		Inducing IL-10 expression [145]
		Blocking NF-kB signaling [146]
		Mediating antiviral signaling [147]
		Promoting cell cycle arrest [81,148]
	NSm	Maintaining mosquito vector infection [88,150,151]
		Role in antiapoptotic function [152,155,156]
		Potential role in neuro-invasion [157]
		Potential role in protein trafficking [157]
		Potential role in mRNA nuclear transport [157]

## Data Availability

Not applicable.
